#  Tophaceous gout of the atlantoaxial joint: a case report

**DOI:** 10.1186/s13256-020-02638-9

**Published:** 2021-02-15

**Authors:** Andrew Benjamin Romero, Evan Paul Johnson, John S. Kirkpatrick

**Affiliations:** 1 Department of Orthopaedic Surgery, Orlando VA Medical Center, Orlando, FL 32827 USA; 2 University of Central Florida College of Medicine, 13025 Cordelia Lane, Apt 301, Orlando, FL 32824 USA

**Keywords:** Gout, Radiculopathy, Neck pain, Cervical spine, Atlantoaxial joint, Tophaceous, Spine

## Abstract

**Background:**

To report the occurrence of tophaceous gout in the cervical spine and to review the literature on spinal gout.

**Case presentation:**

This report details the occurrence of a large and clinically significant finding of tophaceous gout in the atlantoaxial joint of the cervical spine in an 82-year-old Caucasian man with a 40-year history of crystal-proven gout and a 3-month history of new-onset progressive myelopathy. The patient's American College of Rheumatology/European League Against Rheumatism (ACR/EULAR) criteria score was 15.0.

**Conclusion:**

Spinal gout is more common than previously thought, and it should be considered in patients who present with symptoms of myelopathy. Diagnosis can be made without a tissue sample of the affected joint(s) with tools like the ACR/EULAR criteria and the use of the “diagnostic clinical rule” for determining the likelihood of gout. Early conservative management with neck immobilization and medical management can avoid the need for surgical intervention.

## Background

Gout arthropathy is the most prevalent form of inflammatory arthritis in men, wherein a supersaturated blood concentration of uric acid (hyperuricemia) secondary to overproduction and/or under-excretion commonly leads to the precipitation of monosodium urate (MSU) crystals within and around joints, triggering an immune response [1]. MSU crystals can also form within connective tissues, creating what is known as a tophus [1].

Though MSU crystal deposition within and around spinal joints is rare, multiple authors have suggested that it is more common than previously thought [2–4]. Tophaceous gout of the spine was first described by Kersley *et al.* in 1950, when a postmortem process was identified in the atlas of the cervical spine, leading to a forward subluxation of atlas over axis [5]. Following this initial report, a comprehensive review conducted in 2015 revealed 131 total cases involving the spine: 32 cervical, 23 thoracic, 49 lumbar, 18 lumbar and sacral, one cervical and thoracic, and six thoracic and lumbar; two cases did not mention where gout had affected the spine [6]. MSU crystals have been previously described in many spinal structures including the vertebral bodies [7], facet joints [3, 8], pedicles [8, 9], intervertebral discs [10, 11], ligamentum flavum [12], and the epidural space [3]. Very rarely has tophaceous gout been described of the atlantoaxial joint, with only six cases reported to date [4, 5, 13–17]. This report adds the seventh. Previous reports have shown involvement of the transverse process of atlas [5], anterior arch of atlas [4, 17], and odontoid process of axis [4, 13–16] with subsequent subluxations [5, 14–16] or cord compressions via mass effect [13, 17].

## Case presentation

An 82-year-old Caucasian man presented to clinic with new-onset bilateral hand numbness and paresthesia over the course of 3 months. His medical history was significant for a 40-year history of crystal-proven, chronic gout managed with 100 mg allopurinol per day, diabetes mellitus, and coronary artery disease complicated by atrial flutter and managed with an automatic implantable cardioverter-defibrillator. His last gout flare was 1.5 years prior to presentation. He denied changes to his gait, but he used a walker and wheelchair for mobility. His reported ability to stand was unchanged. Previous notes in his medical record indicated that his symptoms had been progressive. He reported inability to use eating utensils or complete other fine motor movements, along with bilateral loss of strength in the upper extremities and periodic suboccipital neck pain. He denied fever, chills, weight loss, and incontinence.

On exam, he presented with full range of motion of his head, neck, and upper extremities. Tophi were observed on his hands, elbows, and metatarsophalangeal joints. Interosseous atrophy was observed in his hands. Light touch sensation was diminished in all dermatomes in his hands and feet following a “stocking-glove” pattern. A graded motor exam of the upper extremities revealed 4/5 strength of both the right deltoid and bilateral finger abductors. No other upper extremity motor deficits were observed. Reflexes were equal bilaterally, and Hoffman’s response was absent. Of note, his knee jerk reflexes were 3+, while his ankle jerk reflexes were 0. His gait was not tested, but he was noted to have imbalance upon standing. Graded motor exam of the lower extremities revealed 4/5 strength of his hip flexors and 3/5 strength of his knee flexors and extenders; his ankles were unable to be tested due to immobility from severe arthritis. His extensor hallucis longus was functional bilaterally, but a graded motor exam could not be reliably assessed due to stiffness of his metatarsophalangeal joints from tophi. Laboratory studies revealed normal uric acid (5.7 mg/dL; normal range: 3.5–7.2 mg/dL) and C-reactive protein (0.449 mg/dL; normal < 0.5 mg/dL), but abnormal estimated glomerular filtration rate (26 mL/min; normal > 60 mL/min) and serum creatinine (2.4 mg/dL; normal range 0.7–1.2 mg/dL) (Table 1).

**Table 1:** Laboratory data over time

Time since presentation (months)	eGFR (mL/min)	Cr (mg/dL)	UA (mg/dL)	HbA1c (%)
0	26	2.4	5.7	7.2
1	53	1.3	4.5	N/A
6	> 60	0.9	4.8	6.5
12	> 60	1.2	4.7	6.7

*eGFR* estimated glomerular filtration rate, *Cr* serum creatinine, *UA* serum uric acid, *HbA1C* hemoglobin A1c, *N/A* not available

**Fig. 1:**
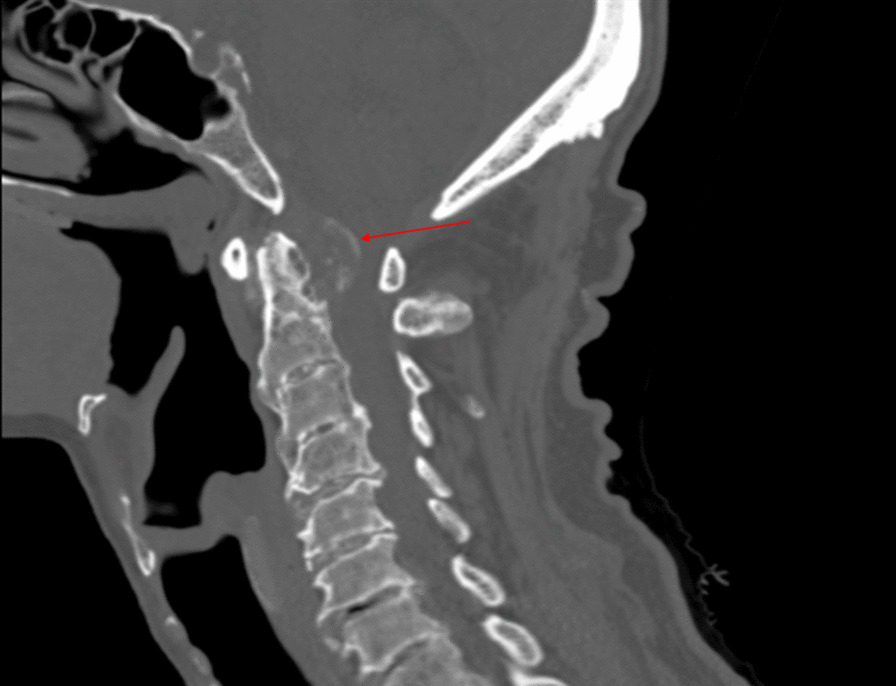
Sagittal computed tomography of cervical spine revealing a large, retrodental calcific mass following the contour of the transverse ligament of atlas, causing severe spinal canal stenosis. The radiographic finding is highlighted by the arrow

**Fig. 2:**
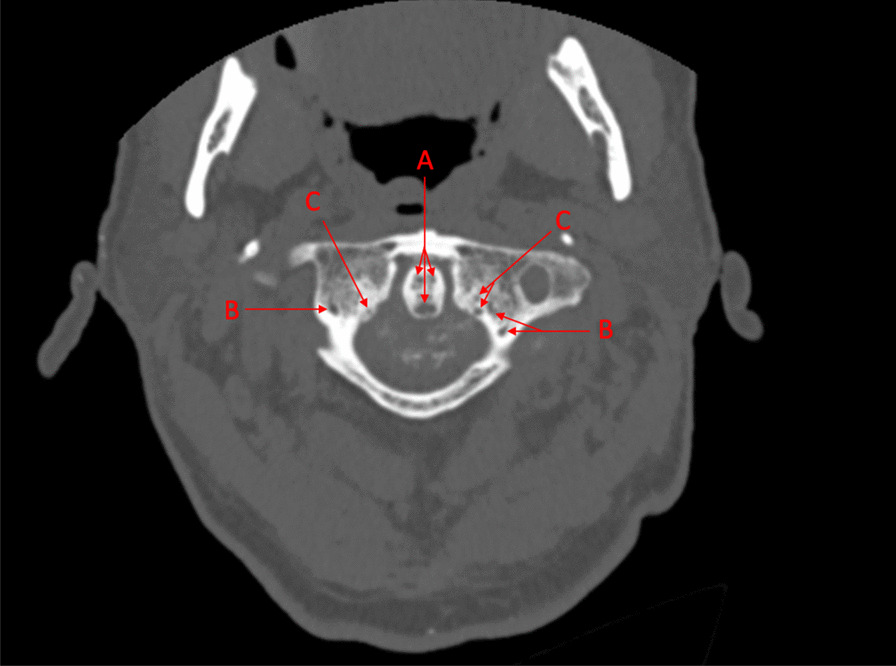
Transverse computed tomography of atlantoaxial joint with noticeable involvement of the odontoid process of axis (**a**), the lateral masses of atlas (**b**), the tubercles of atlas (**c**) for the transverse ligament, and the transverse ligament itself

Computed tomography (CT) of the cervical spine revealed significant arthritis with ankylosis of the mid-cervical spine. Atlas and axis revealed rat-tooth erosions with a large retrodental mass appearing to arise from the transverse ligament of the atlas, containing calcific deposits and causing cord compression, with approximately 80% spinal canal stenosis (Figs. 1, 2). The radiologist advocated that the CT depiction was consistent with gout spondyloarthropathy, especially in the context of the patient’s history.

With the radiological findings, symptomatology, and medical history of the patient, an American College of Rheumatology/European League Against Rheumatism (ACR/EULAR) criteria analysis demonstrated a score of 15.0, supporting the diagnosis of spinal gout; this was shared with the patient. Given the sensitive area of the cervical spine involved and the degree of central canal stenosis, surgical intervention was offered to the patient despite his poor surgical candidacy per his risk assessment. Once completely informed of the risks and benefits of surgery, he refused either surgical or other invasive interventions and tests, including tissue biopsy. His neck was immobilized with a cervical collar, and he was medically managed with a prednisone taper, colchicine 0.6 mg every other day, and allopurinol increased to 300 mg once a day. His symptomatology improved greatly with this regimen. He was referred to both rheumatology and neurology for consult and follow-up. Within the first month, his weakness subsided, and his fine motor coordination returned to baseline. At 6-month and 12-month follow-up, his condition remained stable at the new allopurinol and colchicine dosages. Laboratory data at these follow-up visits, where available, are provided in Table 1. Unfortunately, he was admitted to hospice care with an ejection fraction of 19% the year following our initial visit, and his comorbidities eventually lead to his death a few months later.

## Discussion

Since it was first described, tophaceous gout of the spine has been reported in an increasing number of cases. It is important to recognize that spinal gout is likely more common than originally thought, and it should be included in the differential diagnosis of patients with myelopathies. Advanced imaging techniques, when combined with history and physical exam, are often enough to make a noninvasive, unconfirmed diagnosis of gout [4, 17]. Tissue sample under light microscopy remains the gold standard for proven diagnosis and should be performed whenever possible [18]. When a patient presents with neurological symptoms, and a pathological lesion can be identified on imaging, the patient should undergo surgical amelioration as appropriate per risk assessment to stabilize the spine and decompress the spinal cord. When a patient lacks severe symptomatology inhibiting their baseline daily function, conservative management is more appropriate [2].

In this case, we were unable to obtain a tissue sample due to both the wishes of the patient and the anatomically difficult locus of gouty involvement. However, multiple tools do exist for the diagnosis of gout in a patient who refuses or is a poor candidate for joint or tissue sampling [18–20]. A Dutch research team introduced a logistic regression of seven independent predictors of gouty arthropathy, which yielded a checklist-scorecard that offers 80% positive predictive value and 97% negative predictive value when used to make a clinical diagnosis [19, 20]. Their tool, which they describe as a “diagnostic clinical rule” for determining the likelihood of gout, indicates that the symptoms are due to gout when the points sum to a score greater than or equal to 8.0 [19, 20]. This patient’s score was 8.0 for his history of cardiovascular disease, male sex, previous history, and involvement of the first metatarsophalangeal joint. Additionally, the ACR/EULAR collaborative initiative in 2015 released criteria with sensitivity and specificity of a diagnosis supported by biopsy of 0.92 and 0.89, respectively, versus 0.85 and 0.78, respectively, for a purely clinical diagnosis [18]. The ACR/EULAR criteria have a maximal score of 23.0, and any score greater than or equal to 8.0 is sufficient for diagnosis of gout [18]. This patient scored 15.0 points per ACR/EULAR criteria. Combined, these diagnostic tools, the radiologic findings, the patient’s medical history, and the patient's myelopathic symptom improvement following medical therapy support the diagnosis of spinal gout in this patient.

A point of consideration, as outlined by Petreski *et al.*, is the possibility of a lower threshold of hyperuricemia in a gout patient who also has poor renal function. Their article, titled “Hyperuricemia, the heart, and the kidneys—to treat or not to treat?,” describes how the use of urinary sediment analysis for the presence of urate crystals may be an alternative mode of diagnosing hyperuricemia. The patient in the current report, who undoubtedly had some element of cardiorenal syndrome in his clinical picture, as evidenced by his poor cardiac function and elevated creatinine at presentation, may have had functional hyperuricemia secondary to his diminished ability for uric acid clearance. This possibility, when coupled with his improvement following treatment from both a renal standpoint and an overall clinical standpoint, supports further investigation in this regard [21].

Spinal gout of the atlantoaxial joint is exceedingly rare. Patients who are found to have gout of the atlantoaxial joint should be treated carefully due to the criticality of the area. Previous cases of atlantoaxial joint gout with odontoid involvement have reported success with both conservative and invasive management [4, 13–16]. One case reported success with neck immobilization via Philadelphia collar followed by medical therapy [15]. Others have demonstrated surgical intervention with occipitocervical fusion as an appropriate method of stabilization, still followed by medical therapy [13, 14]. Benefits of surgical intervention include the immediate decompression of the spinal cord and stabilization of the atlantoaxial joint to prevent progression of function loss. Although there was a similar pathological presentation in these cases, surgical and nonsurgical modes of neck stabilization followed by medical therapy provided the same clinical result. Ultimately, the correct course of action depends on the physician’s best judgement, the degree of severity of the gouty arthropathy, and respect for patient autonomy. In the case of the patient in the current report, the criticality of the area of involvement and progressive, severe symptomatology led the medical team to initially offer the patient surgical intervention to both decompress the spinal cord and stabilize the spine in an effort to preserve remaining function and prevent further deterioration.

## Conclusions

Tophaceous gout of the spine is more common than previously thought, and it should be considered in patients presenting with myelopathies, especially those with a history of crystal-proven gout. When obtaining a tissue sample is anatomically difficult and/or the patient does not consent to tissue sampling, diagnosis can still be made with either the diagnostic clinical rule for determining the likelihood of gout or the ACR/EULAR criteria. Diagnosis is further supported by pathognomonic radiographic findings.

Treatment of spinal gout of the cervical spine should include neck immobilization via brace or fusion surgery followed by medical therapy. Given that similar outcomes have been reported for both surgical and brace stabilization of the cervical spine for cases of atlantoaxial spinal gout, we suggest that practitioners consider conservative management with bracing before resorting to surgical decompression in the treatment of gout of the cervical spine.

## Data Availability

Not applicable.

## Abbreviations

MSU: Monosodium urate
CT: Computed tomography
ACR/EULAR: American College of Rheumatology/European League Against Rheumatism
eGFR: Estimated glomerular filtration rate
Cr: Serum creatinine
UA: Serum uric acid
HbA1C: Hemoglobin A1c
